# Heterogeneity of Scaffold Biomaterials in Tissue Engineering

**DOI:** 10.3390/ma9050332

**Published:** 2016-05-03

**Authors:** Lauren Edgar, Kyle McNamara, Theresa Wong, Riccardo Tamburrini, Ravi Katari, Giuseppe Orlando

**Affiliations:** Wake Forest School of Medicine, 1 Medical Center Blvd Winston-Salem, Winston-Salem, NC 27157, USA; laedgar@wakehealth.edu (L.E.); kmcnamar@wakehealth.edu (K.M.); twong@wakehealth.edu (T.W.); rtamburr@wakehealth.edu (R.T.); rkatari@wakehealth.edu (R.K.)

**Keywords:** biomaterials, extracellular matrix scaffold, decellularized organs, natural polymer, synthetic polymer

## Abstract

Tissue engineering (TE) offers a potential solution for the shortage of transplantable organs and the need for novel methods of tissue repair. Methods of TE have advanced significantly in recent years, but there are challenges to using engineered tissues and organs including but not limited to: biocompatibility, immunogenicity, biodegradation, and toxicity. Analysis of biomaterials used as scaffolds may, however, elucidate how TE can be enhanced. Ideally, biomaterials should closely mimic the characteristics of desired organ, their function and their *in vivo* environments. A review of biomaterials used in TE highlighted natural polymers, synthetic polymers, and decellularized organs as sources of scaffolding. Studies of discarded organs supported that decellularization offers a remedy to reducing waste of donor organs, but does not yet provide an effective solution to organ demand because it has shown varied success *in vivo* depending on organ complexity and physiological requirements. Review of polymer-based scaffolds revealed that a composite scaffold formed by copolymerization is more effective than single polymer scaffolds because it allows copolymers to offset disadvantages a single polymer may possess. Selection of biomaterials for use in TE is essential for transplant success. There is not, however, a singular biomaterial that is universally optimal.

## 1. Introduction

Organ and tissue transplantation are used prolifically to treat a multitude of diseases and wounds. Though transplantation represents a successful and life-saving treatment modality, the supply of donor grafts has not kept pace with the growing demand from end-stage patients. In the past decade, the paucity of transplantable organs has increased along with the discarding of potential grafts further augmenting the gap between supply and demand [1]. Moreover, those who receive transplants are at risk for transplant rejection and complications from immunosuppression [2]. Approaches in regenerative medicine, however, may pave the way for a potentially inexhaustible source of transplantable organs that would not require immunosuppression following transplant [3,4].

**Figure A1:**
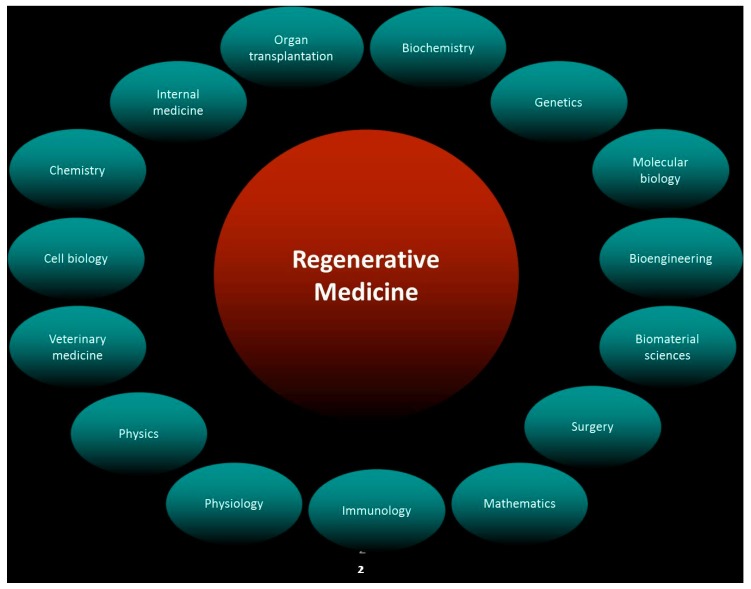
Regenerative medicine is a discipline with contours still being defined. By nature, it incorporates and leverages numerous fields of health sciences, as illustrated in the figure below which is extrapolated, with permission, from Orlando G *et al.* *Gut*, 2013 [148].

Tissue engineering (TE) is a closely related subfield of regenerative medicine (Figure A1) that focuses on the repair, replacement, and regeneration of tissues and organs with impaired function [3,5,6]. TE offers the ability to synthesize tissues with unique properties to create specific tissues [7]. Common strategies involve the use of porous 3D scaffolds which serve as templates for specific tissue formation [8]. These templates are seeded with cells such as embryonic stem cells or adult stem cells along with growth factors to stimulate cell growth mechanically or chemically [8,9]. Scaffolds may also be used as injectable or transplantable implants to enhance reparative pathways of the human body or to facilitate the delivery of non-cellular biomaterials, like drugs and other bioactive compounds into the body.

A variety of biomaterials has been studied in the quest for the ideal scaffolds. Several factors are highly significant to the success of materials used in TE. Ideally, a biomaterial should be biocompatible, biomimetic, non-toxic, and non-immunogenic [10]. Immunogenic materials that elicit inflammation hinder vascularization and remodeling, which are essential to graft efficacy and survival [11]. The most successful scaffold materials facilitate cell adhesion, growth, migration, and respond to molecular and physical cues of the *in vivo* milieu [12,13]. Biodegradability of materials is a factor specific to the desired function of the tissues engineered. Slow biodegradation is preferred for long-term implants, whereas active and rapid biodegradation is important to encourage remodeling and replacement of biomaterials for the purposes of tissue repair [12].

In this review, these criteria will be applied to analyze the efficacy of the following materials as scaffolds used in TE: natural polymers, synthetic polymers, and extracellular matrix (ECM)-based scaffolds from decellularized animal or human organs.

## 2. Geometry and Delivery

The geometrical and anatomical configuration of a given scaffold can be intentionally manipulated to optimize delivery of cells or bioactive material to the target tissue or organ. Specific scaffolds can also be chosen to provide appropriate signals to the seeded cells necessary for adhesion, proliferation, and/or differentiation.

### 2.1. Structure

#### 2.1.1. Simple Structure

Some of the earliest attempts at creating biocompatible scaffolds were based on simple structures with flat two-dimensional (2D) geometries. The applications for 2D grafts have been widely studied in the field of skin engineering. Numerous synthetic skin grafts already exist in the marketplace and offer replacement of epidermal layers, dermal layers, or a combination of the two [13]. The majority of these grafts are composed of scaffolds seeded with fibroblasts and/or keratinocytes. The simple structure of these grafts, however, has negatively affected the vascularization of these skin grafts. Therefore, attention is now turning toward ways to incorporate more complex geometry into the grafts to accommodate better blood supply to the transplanted region.

More complex two-layered scaffolds have been developed for the purpose of engineering vasculature and more tubular structures. One application has been found in urethral engineering in which numerous studies have sought to create scaffolds with an inner layer of epithelial cells and an outer layer of muscle cells. Similar techniques have been employed in vascular engineering with fibroblasts and endothelial cell layers [14].

#### 2.1.2. Three-Dimensional (3D) Structure

3D structures remain a large challenge for researchers in part because of the complex organization of different cell types within each organ system. To replicate complex structures, research has focused on two techniques: bioprinting and decellularized organs. The idea of bioprinting organs has only existed for two decades and revolves around the idea of using a 3D printer to create a proper scaffold on which to seed multiple cell layers to produce a fully functioning organ [15]. 3D bioprinting, when compared to non-biological printing, involves additional complexities, such as the choice of materials, cell types, growth and differentiation factors, and technical challenges related to the sensitivities of living cells, the construction of tissues and the “microscopy scale”. 3D bioprinting has already been used for the generation and transplantation of several tissues, including multilayered skin, bone, vascular grafts, tracheal splints, heart tissue and cartilaginous structures. Other applications include developing high-throughput 3D-bioprinted tissue models for research, drug discovery and toxicology, as recently reviewed by Murphy and Atala [16,17].

On the other hand, ECM scaffolds derived from the decellularization of native organs seem to offer the quickest route to clinical application, because they are biocompatible *per se*, and preserve basic components (proteins and polysaccharides, matrix-bound growth factors, and cytokines). In addition, they retain an intact and patent vasculature capable of sustaining physiological blood pressure when implanted *in vivo* and can drive differentiation of progenitor cells into an organ-specific phenotype [18,19,20,21]. Furthermore, because they are derived from native organs, appropriate three-dimensional structure is retained.

### 2.2 Composition

#### 2.2.1. Sponge

Sponge-based scaffolds are composed of interconnected micropores with notable fluid absorption and hydrophilicity [22]. Furthermore, their mechanically weak architecture, pliability, and degradability make them advantageous as potential vehicles for wound repair [23]. One animal study using chitosan-gelatin sponge wound dressings found improved antibacterial properties and decreased risks of hypertrophic scar formation in comparison to gauze wound dressings [24]. Another animal model study performed with triphala incorporated collagen sponges demonstrated increased wound closure rates and decreased bacterial colonization as compared to gauze [25]. Although early animal studies have shown promising results, human trials using gentamicin-collagen sponges have had mixed results. A randomized trial of cardiac surgery patients found no significant difference in wound dressing infection incidence between wounds closed with sponge and wounds closed without sponge [26]. A more recent study in diabetic patients undergoing amputations, however, found a significant increase in the rate of wound closure without infection when a gentamicin-collagen sponge was used [27].

#### 2.2.2. Mesh

Mesh-based scaffolds have gained traction in TE as a potential source for cartilage tissue regeneration because of their ability to mimic the natural structural environment of soft tissue. Mesh scaffolds are created using electrospun nanofibers composed of the biomaterial polymer of interest and are woven into a 3D structure creating an environment for cellular adhesion and growth. Analysis of mesh scaffolds has shown uniform porous structures with diameters similar to naturally occurring extracellular matrix (ECM) [28]. Pretreatment of fibrous polyglycolic acid mesh scaffolds with adipose-derived stem cells were used in one study to analyze the properties of articular cartilage repair in femur trochlea [29]. This study found a more representative cellular composition in the pretreated models as compared to controls treated with only the scaffold. The composites also restored the compressive moduli of the cartilage to 88% of normal cartilage by 6 months post implant, as compared to 50% at 3 months. Further research emphasizes the importance of creating meshes with the right balance of mechanical and bioactive properties. One study performed using chitosan/PCL mesh scaffolds found that mechanical properties improved as the ratio of PCL increased, but that neo-cartilage formation was favored with higher chitosan ratios [30]. This highlights the challenge of balancing immediate function with long-term integration in cartilage engineering.

#### 2.2.3. Hydrogel

Hydrogel-based scaffolds exhibit a wide range of applications in part because of their more pliable structure and composition [31]. Hydrogels are composed of crosslinked natural or synthetic polymers in a gel-like consistency that can be injected into the human body [32]. This property led to applications of hydrogels in the research of angiogenesis, bone repair, and cartilage regeneration [33]. A further advantage of hydrogels is the ability to change their degradation rates by changing the number of crosslinks between polymers, allowing for more targeted use in TE [34]. This has been demonstrated *in vivo* with animal studies that found adjustable rates of degradation in polyethylene glycol hydrogels used for cartilage repair [35]. More recent research in the field has focused on adjusting mechanical properties of hydrogels to control rate of cell proliferation. Studies in animal models have found that adjusting the mechanical properties of the hydrogel modified the attachment rate of chondrocytes and fibroblasts seeded on the hydrogel [36]. Other animal models have found improved chondrocyte morphology and integration with PLGA/chondroitin/hyaluronate hydrogel scaffolds [37]. Additionally, hydrogels offer a drug-delivery platform that can attract and nourish growing cells as they integrate [38]. This has been used in animal models to deliver TGF-beta growth factors to promote collagen type II proliferation in cartilage repair [39]. Further research has looked to add arthritis drug delivery to cartilage-promoting hydrogels in an attempt to reverse the effects of rheumatoid arthritis while offering simultaneous relief of symptoms [40].

#### 2.2.4. Particle-Based Constructs

Cell proliferation and differentiation can be enhanced via growth factor delivery using nano- and micro-particle constructs. Isogai *et al.* found that although basic fibroblast growth factor (b-FGF) can augment chondrogenesis in solution, 97% of it dissociates from the site of implantation within the first 24 h [41]. They were able to sustain the release of b-FGF over time by incorporating it into gelatin microspheres. This allowed for more managed release of the growth factor that retained 18% of the initial amount 2 weeks post implant. Another study using synthetic PLGA microspheres showed that bone morphogenic proteins were better retained at the site of implant in rodent calvarial bones and resulted in more than triple the thickness, whereas a hydrogel model increased thickness by only 66% [42].

The use of nanoparticles extends beyond drug delivery techniques. Research has also indicated nanoparticles as a potential source of self-assembling scaffold material that can be injected into wound sites much more easily than pre-formed ECM implants. Liu *et al.* used star-shaped poly(l-lactic acid) microspheres with a diameter of 160 nm that were able to self-assemble into a structural matrix conducive to cartilage regeneration in rabbit models [43]. Similarly, other researchers have found success using a phenylalanine ethyl ester substituted polyphosphazene polymer with 100 nm-sized hydroxyapatite to create 3D porous scaffolds with good osteoblast cell adhesion, proliferation, and alkaline phosphatase expression [44]. The resultant scaffold also demonstrated compressive moduli of 46–81 MPa and demonstrated promising results for bone TE.

## 3. Natural Polymers

Naturally occurring polymers are a subset of bioactive materials that may be used in the human body for TE applications. These can be grouped into three categories: polysaccharides, proteins, and nucleic acids [45]. As naturally occurring compounds, these materials have favorable compatibility profiles *in vivo*, both theoretically and empirically. Thus, they are better suited to overcome issues of toxicity or antigenicity.

There are, however, foreseeable disadvantages to the use of bioactive materials. In the case of autoimmune disease processes, for example, creating materials identical to the lost biological component may not work. Instead, the immune system could potentially recognize the biomaterial implant as identical to the original antigenic tissue. In addition, bioactive materials are not as long lasting as their synthetic counterparts and will often degrade over time. This can be advantageous because modifications to the structures can control the rate of degradation to suit therapeutic needs [46]. Therefore, bioactive materials must be created with the purpose of eventually restoring the physiologic equilibrium of the host or must be re-administered. From a manufacturing standpoint, the composition of bioactive materials is much more complex than their synthetic counterparts [47]. Of course, this feature is also the reason that bioactive materials are able to mimic *in vivo* environments better. The characteristics and clinical applications of a sample of these bioactive materials are provided below.

### 3.1. Polysaccharides

#### 3.1.1. Cellulose

Easily derived from a wide variety of plants, cellulose is the most abundant polysaccharide used in TE applications [48,49]. Its structure, consisting of repeating beta-d-glucopyranose rings, provides mechanical strength and support to scaffolds [50].

Cellulose nanocrystals have been successfully used in reinforcing polymers with limited tensile strength. Anisotropic cellulose nanofillers augment mechanical strength of ECM scaffolds allowing for bioengineering of load-bearing tissues, specifically tendons and ligaments [51]. Cellulose also lends itself well to the more rigid tissues of the human body such as bone, cartilage, and cardiac tissues [52,53,54].

To prime cellulose for bio-integration, it is often necessary to create composites of cellulose with other materials. With regard to bone TE, previous research has demonstrated significantly improved rates of hydroxyapatite growth with the phosphorylation of bacterial cellulose [55]. Similar results have been shown in citric acid and calcium phosphate-modified cellulose [56,57]. This improved on previous research, which had encountered immunogenicity when introducing unmodified cellulose to hydroxyapatite [58].

#### 3.1.2. Chitin and Chitosan

Chitin is the second most abundant natural polysaccharide after cellulose and is derived from crustacean exoskeletons, giving it a natural tendency to dictate shape and form [59]. Its structure consists of a linear beta 1,4-linked *N*-acetyl-d-glucosamine, creating hydrophobic chains [60]. Although these hydrophobic interactions may limit the utility of a biomaterial, intrinsic dielectric properties of chitin render it useful in TE of tissues requiring electrical conductance, such as nerves.

Because chitin possesses the electrical conductivity that neuronal repair and regeneration requires, chitin has significant application in the treatment of neurodegenerative diseases. Furthermore, the chitin can be modified to augment its electrical conductivity. Recently, the addition of carbon nanotubes to a chitin scaffold was shown to increase in electrical conductivity, synaptic function, and viability of engineered neurons [61].

Chitosan has demonstrated antibacterial properties and is already used to promote wound healing in veterinary medicine [62,63]. Research has also shown it to have some efficacy with tissue regeneration in the setting of periodontitis [64]. To improve the bioactivity of chitosan, research has looked to modify the polymer through copolymerization. One such study found increased swelling capacity and increased hemocompatibility of chitosan when copolymerized to acrylic acid and 2-hydroxyethyl methacrylate [65]. Another study found that subjecting chitosan to *N*,*O*-carboxymethylation increased the antibacterial properties of chitosan [66]. For these reasons, chitosan is a common polysaccharide used in bone and skin TE.

#### 3.1.3. Hyaluronic Acid

Hyaluronic acid is a glycosaminoglycan originally derived from bovine vitreous humor and consists of *N*-acetyl-d-glucosamine and beta-glucuronic acid [67]. Hyaluronic acid is much heavier than most glycosaminoglycans and exhibits a molecular weight that often ranges between 1 kDa and 10 MDa [68]. High molecular weights and intramolecular forces give hyaluronic acid a high viscoelasticity that has already proven useful in joint repair because of its simultaneous ability to lubricate and to shock absorb [69].

Many studies have modified the carboxyl, hydroxyl, and acetamido groups on hyaluronic acid, via acrylation [70]. These modified forms of hyaluronic acid have been used in conjunction with bone growth-promoting factors such as bone-morphogenic protein-2 and human mesenchymal stem cells to promote bone growth *in vivo* [71]. Hyaluronic acid is naturally degraded by hyaluronidase *in vivo*, which more recent studies have sought to use to advantage by modifying the rate of degradation to optimize bone growth [72].

### 3.2. Proteins

#### 3.2.1. Collagen

Collagen is an integral component of a wide range of tissues, which not only inures to its biomimeticity as an ECM scaffold material but also allows for its diverse application in TE [73]. Unlike polysaccharides, the structure of collagen is much more variable among species [74,75]. Different forms of collagen are present in particular tissues permitting greater selectivity in collagen biomaterial. The four most common forms include: type I, which is found in skin, tendons, and bone; type II, which is found in cartilage; type III which is found in blood vessels; and type IV, which is found in basement membranes [76]. Variation in collagen form, in turn, allows for enhanced biomimeticity because one can selectively use the type of collagen that is specific to the end-target tissue.

For example, scaffolds made of type I collagen exhibit contractility, which occurs as part of osteogenesis in native tissues. Recently, it has been demonstrated that increased contraction of type I collagen enhances repair and regeneration of bone with specific application in the treatment of osteochondral lesions and osteoarthritis [77]. Conversely, the resistance to contraction exhibited by type II collagen offers a potential solution to the problem of maintaining shape of engineered tissues. In particular, use of a type II collagen scaffold prevented shrinkage and shape change of tissue-engineered ear cartilage implants [78].

By crosslinking different types of collagen to natural or synthetic polymers, scientists have already demonstrated a vast array of TE applications. Some recent applications for skin TE include type I collagen-alginate scaffolds and 3D collagen scaffolds that attempt to mimic better natural architecture [79,80]. 3D collagen scaffolds have also been used with type II collagen to create scaffolds for cartilage tissue [81]. Certain areas of tissue regeneration, such as tendon bioengineering, are particularly amenable to collagen-based scaffold manipulation [82]. Recent research studies have also shown evidence of the growth of tendon tissue on collagen-glycosaminoglycan scaffolds *in vitro* [83]. Future applications of collagen bioengineering are focused on nerve regeneration. Already the use of type I and type III collagen electrospun scaffolds has demonstrated promising results *in vitro* [81].

#### 3.2.2. Elastin

Elastin is a major contributing protein to the extracellular matrix of various tissues including arteries, lungs, ligaments, and skin [84]. As the name suggests, elastin is largely responsible for the elastic properties inherent in these tissues by virtue of its numerous hydrophobic domains (predominately consisting of Gly, Val, Pro, and Ala) and crosslinked tropoelastin molecules [85].

Furthermore, elastin is remarkable for its longevity in the human body. As a main component of the ECM, it undergoes constant cyclic loading forces and yet shows no appreciable deterioration or turnover [86]. The combination of these properties makes elastin another desirable bioactive material for TE research and biomaterial development.

Although elastin has technically existed as a biomaterial for quite some time in the form of autografts, the advent of decellularization and recellularization has added new applications of elastin as an organ transplant biomaterial. Studies have shown that the cellular material of tissues can be removed via detergents without affecting the elastin component or the mechanical properties of the ECM [87]. This has allowed researchers to remove the immunogenic properties of complex organs while preserving their natural architecture such that non-immunogenic material may be added and readily accepted by the organ recipient. This has already been achieved for relatively simple organs such as bladders, heart vasculature, and esophagi [88,89,90]. The application of decellularized matrices, however, is still in its infancy. Further research is still necessary to realize applications for more complex organs such as the heart and the liver [91,92].

Elastin is also useful for the modification of stiff scaffolds lacking viscoelastic properties. Recently, the addition of insoluble elastin to collagen scaffolds was observed to decrease tensile strength and compressive moduli of the scaffold [93]. Further, elastin modification of collagen scaffolds was shown to alter biological response to the scaffold. Smooth muscle cells seeded on elastin-collagen scaffolds displayed a contractile phenotype marked by a decrease in proliferation, whereas they exhibited a synthetic phenotype with increased proliferation when seeded on collagen-only scaffolds [93]. The ability of elastin to modulate the mechanical properties of and biological response to certain biomaterials provides the opportunity to construct highly dynamic composite scaffolds.

## 4. Synthetic Polymers

Synthetic polymers are highly tunable macromolecules that can be used to form scaffolds for use in TE. Polyesters, polyether esters, polyurethanes, and attenuated silks are some of the most prolific synthetic materials used in bioengineering. Synthetic polymers lack biological signaling capability and, therefore, require conjugation with molecules and cellular markers [94]. Nevertheless, synthetics are beneficial because they can be specifically tailored to possess desired properties of certain scaffolds. Chemical and mechanical properties are easily altered to accommodate and encourage growth of specific tissues by the addition of cell-adhesion molecules, moieties, and functional groups [12,95,96].

Relative to natural polymers, synthetics have limited immunogenicity and reduced bioactivity, which decrease risk of rejection *in vivo*, but have greater potential to evoke inflammatory responses as compared to other biomaterials [8]. Degradation products of synthetic polymers create an acidic pH localized to the site of implantation which instigates tissue inflammation that can potentially induce subsequent fibrosis [97]. Synthetics remain advantageous because they are easily manufactured at relatively low cost [95]. Specific properties and applications of prevalent synthetic polymers are discussed below.

### 4.1. Polyesters

Polyglycolic acid (PGA), polylactic acid (PLA), and polycaprolactones (PCL) are the most prevalent polyester biomaterials. Polyesters have been used successfully in the development of numerous tissues, but unfortunately have poor cellular recognition that limits differentiation of attached cells [96,97].

A variety of polyester scaffolds have been used, including single polymer scaffolds and copolymer scaffolds. Copolymerized scaffolds have greater efficacy because they allow for properties unique to certain polyesters to be combined to complement one another. Zant *et al.* demonstrated that tensile strength, in particular, was maximized when PDLLA, PEG, and poly(1,3-trimethylene carbonate) were photo-crosslinked [98].

Among synthetic biomaterials, polyesters pose the greatest risk, however, of an inflammatory response. Their degradation results in acidic byproducts that produce inflammation and granulation. In addition to evoking an inflammatory response, acidic byproducts perpetuate themselves by further breaking down the graft scaffold [99]. Subsequent fibrosis reduces vascularization, graft survival, and eventually causes graft necrosis [97].

#### 4.1.1. Polyglycolic Acid (PGA)

PGA exhibits rapid degradation *in vivo* rendering it an ideal material for mesh network scaffold. PGA is most commonly used in TE as a mesh form in which seeded or native cells can quickly replace the scaffold [100]. Jacobs *et al.* demonstrated that this transient mesh network is ideal for laryngotracheal reconstruction [101]. Rapid degradation, however, reduces mechanical strength of PGA and renders it an unsuitable material for TE of tissues that require high load-bearing capacity.

#### 4.1.2. Poly-Lactic Acid (PLA)

PLA is a more hydrophobic molecule than PGA making it more stable and more resistant to degradation by hydrolysis [100]. PLA has several forms due to its chirality. These forms include L and D enantiomers, and a mix thereof: poly(l-lactic acid) (PLLA), poly(d-lactic acid) (PDLA), and poly(d,l-lactic acid) (PDLLA) [99,102]. Of these forms, PLLA and PDLLA are the most commonly used in TE [98]. PLLA exhibits excellent biomimeticity and is the most biocompatible because it is a naturally occurring polymer [103]. Combined with its high porosity, PLLA is particularly efficacious for a high degree of cell attachment and proliferation [12]. PLLA has a relatively slow rate of degradation making it useful in TE of tissues that require long-term high tensile strength such as ligaments and tendons [104]. In addition, PLLA complex with chitosan has exhibited even slower degradation allowing it to serve as guided membranes for tissue regeneration [105]. PDLLA has similar properties to PLLA, such as slow rate of degradation, but has specifically been shown to create a network of indiscriminately distributed fibers useful for the development of keratinocytes and skin grafts [106].

#### 4.1.3. Poly-l-Glutamic Acids (PLGA)

PLGA is a homogeneous random mixture of PLA and PGA. Copolymerization of these molecules is advantageous because both have very different properties that complement each other. PLGA is an amorphous scaffold that is optimal for very complex structures [99]. Currently, it is the most utilized synthetic in 3D printing modalities. It has been used to synthesize bone, cartilage, tendon, skin, liver, and nerves [99]. PLGA in composite with calcium phosphate has particular application for bone regeneration [107]. In addition, PLGA exhibits limited fibrosis upon degradation which makes it an excellent candidate for articular cartilage replacement and regeneration [12]. Zhang *et al.* found that PLGA scaffolds used to make adipose stem cell-spheroid aggregates showed the ability to develop hyaline cartilage [108]. PGA and PLA copolymerized scaffolds can be tailored for specific types of tissues by selectively using one enantiomeric form of PLA, rather than a racemic mixture. For example, isolated PLLA in composite with PGA has specific efficacy against flexure and steady flow which allows for TE of tissue that will be under high stress. PLLA/PGA was used in a recent study to construct heart valves that undergo flex-flow stress [109]. PLLA and PLGA composites have poor cell recognition, however, and demonstrate non-specific signaling which limits the ability of cell differentiation [110].

#### 4.1.4. Polycaprolactone (PCL)

Polycaprolactone (PCL) was the earliest polyester used in TE. Biodegradation of PCL occurs slower than that of PGA or PLA, making it the optimal polyester for development of long-term grafts like bone [100]. It is, therefore, inappropriate for development grafts via tissue replacement of scaffolds by native cells [12]. Degradation products are non-toxic and do not alter local pH. PCL demonstrates increased graft angiogenesis as compared to PLA and PGA whose acidic degradation byproducts prevent sufficient vascularization [30,111]. Although PCL biodegrades well, it is not wholly bioresorbable and its degradation products are not completely removed from the body. This differs from PLA and PGA degradation products which are metabolically active and can be easily converted to CO_2_ and water to be removed by the lungs and kidneys [111]. PCL does evoke a neutrophil response and localized inflammation upon transplant *in vivo*, but, unlike PGA and PLA, inflammation does not result in any long-term adverse effects [112].

### 4.2. Polyether Esters

Polyethylene glycol (PEG) and polybutylene terephthalate (PBT) are the most prevalent polyether esters used in TE. PEG and PBT are mostly found as composite scaffolds. Combination of PEG and PBT is useful because PEG provides elasticity and PBT provides stiffness. Adjustment of PEG:PBT ratios allows for easy manipulation of the scaffold’s structural properties. PEG/PBT scaffolds are easily hydrated, which allows for increased nutrient diffusion through the scaffold, rendering porosity less of a determining factor of tissue growth [113]. In addition, PEG/PBT scaffolds have an increased degree of pore interconnectivity and high surface area for maximal cell adhesion, allowing for the generation of sizeable amounts of tissue. Mendes *et al.* demonstrated that PEG/PBT-based TE produced a large enough amount of bone tissue to support hematopoiesis sufficiently [114]. Specifically, PBT/PEG scaffolds have great utility in bone regeneration relative to other synthetic scaffolds as evidenced by enhanced bone marrow stromal cell adhesion, high level of bone bonding, and calcification *in vivo* [114,115,116].

### 4.3. Polyurethanes

Polyurethanes (PU) are polymers that have recently reemerged as a biomaterial. Originally, PU was not used because its degradation byproduct (2,4-diaminotoluene) was toxic. PU is now widely used in complex with polyhedral oligomeric silsesquioxane (POSS). Conjugation of POSS with PU renders the scaffold non-toxic and non-biodegradable. PU has electroactive and elastic properties similar to soft tissue which promotes cell regulation during TE [117]. In addition, PU is thermoresponsive, mechanically adjustable, and exhibits a high degree of biocompatibility [118,119]. The application of PU has been growing as it is now being used in nanocomposites. In particular, polycarbonate urethanes (PCU) in composite with POSS have great potential for vascular TE. PCU and POSS form a strong and highly viscoelastic nanocomposite that is highly hemocompatible because its surface moieties are fluid at the blood-material interface. Morever, POSS-PCU has anti-thrombogenic properties, rendering it ideal for cardiovascular TE [120,121]. Yahyaei *et al.* found that POSS-PCU scaffolds increase the cell viability of human umbilical vein endothelial cells, which further demonstrates that polyurethanes are excellent materials for vascular TE [122]. In addition, POSS-PCU nanocomposites have been successfully used in engineering cardiovascular bypass grafts, lacrimal ducts, and tracheas [120].

### 4.4. Silk

Silk proteins may arguably be categorized as synthetic or biologic materials. For the purposes of this review, however, silk proteins will be discussed in the context of synthetics. Synthetically derived and synthetically altered silk proteins prove to be more efficacious in terms of biocompatibility as compared to naturally occurring silks [123]. Naturally occurring silks elicit a variety of inflammatory reactions rendering these types of silk suboptimal for graft viability after transplantation. The sericin component of naturally occurring silk is the impetus of the inflammatory response. Sericin can, however, be removed via degumming processing, yielding a synthetically altered silk that is less immunogenic and, therefore, a biomaterial superior to natural silk [124]. In addition, synthetic silks have wider application as a material (scaffolds, gels, and films) than naturally occurring silk that only yields fibers [125]. Intrinsic properties of high tensile strength, flexibility, elasticity, and biocompatibility make synthetic silk a highly effective biomaterial [123].

Synthetic silks convey benefits of natural silk, but with enhanced function. Tokareva *et al.* were able to tailor the function of silk by manipulating its gene-coding regions using DNA recombinant technology. Inserting novel sequences into silk-coding regions takes advantage of the natural property of silks to express molecules coded by said sequences once implanted *in vivo* [125]. Silk also self-assembles which provides opportunity for hybridization of desired molecules to convey a specific function of the scaffold. The bioactive nature of silk, stemming from its natural origin, allows for effective adaptation to environmental cues from native tissues. Successful response to cellular signaling is supported by the recent application of silk fibroin to promote osteogenic differentiation of human mesenchymal stem cells [126].

Silk is also suitable for regeneration of highly vascular tissues. Of note, silk degradation byproducts do not elicit fibrosis, which typically prevents vascularization in other synthetic scaffold-based grafts. Silk exhibits slow degradation *in vivo* which proves beneficial for graft vascularization and native tissue regeneration [127].

## 5. Decellularized Organ Scaffolds

Natural animal or human organs can be decellularized to produce ECM-based scaffolds. Acellular organ scaffolds are highly biomimetic because they retain original structural architecture, vascular networks, adhesion molecules, and markers for cellular signaling [17,18,19,20,21,128,129].

Methods of decellularization can be chemical, physical, or enzymatic. The specifics of the decellularization process are beyond the scope of this review; however, the most commonly used methods in the studies reviewed is perfusion-decellularization with chemical detergents such as sodium dodecyl sulfate (SDS), triton X 100, and sodium deoxycholate. This method is advantageous because it maximizes the delivery of chemical agents and removal of cellular material with minimal damage to the ECM [129].

Early research was conducted using animal-derived (e.g., murine, porcine) organs to explore the integrity of scaffolds formed via decellularization. After effective and functional scaffolds were created in animal models, the area of decellularization expanded to include discarded human organs [130,131]. In the case of kidney TE, for example, approximately 2600 organs annually harvested for transplant are discarded for varying reasons including organ ischemia and anatomic incongruity [132]. Use of discarded human organs as TE scaffolds shows great potential to offset the chronic shortage of donor organs [133,134]. To date, decellularization has been applied to generate skin, intestine, blood vessel, upper airway, liver, kidney, and lung ECM scaffolds.

### 5.1. Heart

TE of a complete heart poses unique challenges because of its high oxygen and energy requirements. A specific geometry of cardiac tissue is necessary to meet such demands, rendering many scaffolds unsuitable. In addition, previously discussed scaffolds do not allow for sufficient perfusion of developing tissue throughout the entire structure. The use of decellularized hearts as a template of TE offers a solution. Whole-heart engineering has been successful in both rat and porcine models in which perfusion decellularization of cadaveric hearts yield scaffolds with preserved fiber composition and orientation. The porcine heart exhibited successful decellularization and recellularization with mesenchymal stem cells [135]. Despite subsequent inflammation and coronary artery thrombosis, heterotopic transplantation was successful. The rat model demonstrated two types of recellularization: perfusion of aortic endothelial cells and intramural injection of neonatal cardiac cells. Recellularized rat hearts were highly anisotropic and lacked evidence of thrombosis or inflammation [136].

### 5.2. Liver

Several studies have applied decellularization techniques to rat and mouse livers that have generated ECM scaffolds with a preserved 3D macrostructure, ultrastructure, ECM composition, microvasculature, bile network, and 50% of original growth factors [92,137,138]. Decellularized livers were able to undergo recellularization to yield functional bioengineered livers, as evidenced by levels of *in vitro* albumin production, urea metabolism, and cytochrome P450 induction comparable to that of a normal liver. More recently, complete and homogeneous decellularization has been achieved. Yagi *et al.* demonstrated the greater clinical application of decellularization by uniformly decellularizing all component segments of a porcine liver [139]. Preservation of the 3D architecture, native and structural basement membrane matrices, growth factors essential for angiogenesis and hepatic regeneration, and functional vascular and biliary networks was observed. Not only was the scaffold structurally sound but it also allowed for high-efficiency engraftment of functional hepatocytes. Recently, human livers have been successfully decellularized with a modified perfusion-decellularization protocol which yielded ECM scaffolds that allowed for efficient homing and targeting of cells to the correct location [140].

### 5.3. Kidney

Kidneys represent one of the most challenging organs for which to generate an ECM scaffold because of their complexity in both structure and function [141]. Bioengineered kidneys must not only maintain the architecture of the kidney but also be able to permit perfusion, filtration, secretion, absorption, and excretion [142]. Despite the challenges, Song *et al.* were able to produce renal ECM scaffolds from rat, porcine and human kidneys with preserved tissue architecture. With complete removal of nuclei and cellular components, recellularization was also possible, resulting in kidneys that maintained perfusion and produced urine for a short time period in a rat model. A similar study achieved success in generating renal ECM scaffolds that maintained innate molecular and spatial framework from discarded human kidneys [132]. These scaffolds also maintained vascular patency and biological function. Recently, rhesus monkey renal ECM scaffolds were studied as potential scaffolding for the generation of human kidneys [141]. Rhesus monkey scaffolds were successfully reseeded with human embryonic stem cells, showing that decellularized organs of a variety of origins have potential in human clinical application.

### 5.4. Lung

Transplantable lungs are at a significant shortage and are frequently damaged during procurement [143]. Although better procedures have been implemented, there is still a gap that can be filled by decellularized lung scaffolds. TE of lungs requires scaffolds that allow for sufficient gas exchange and movement of nutrients and waste to and from the tissues. Song *et al.* produced scaffolds from rat lungs with intact architecture and matrix composition [142]. The alveolar size, volume, and number as well as the rate of gas exchange, vital capacity, and compliance *in vitro* were comparable with native lungs. The scaffold-based lungs, however, did not survive *in vivo* due to severe inflammation and edema post implantation in the rat model. O’Neill *et al.* successfully decellularized porcine lungs, but in comparison to human lung ECM, they lacked mechanical strength and were less metabolically active [144].

### 5.5. Implications for the Future

**Figure A2:**
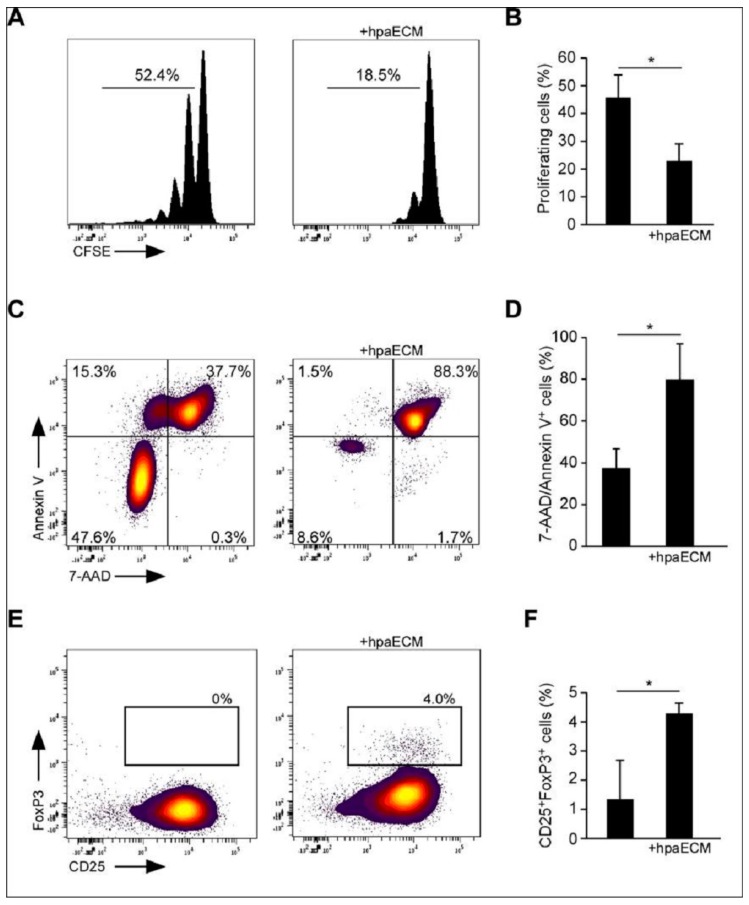
Immune properties of ECM scaffolds obtained from the human pancreas. (**A**) Representative plots and (**B**) quantitation of carboxyfluorescein succinimidyl ester (CFSE) dilution in CFSE-labeled human naïve CD4^+^ T cells stimulated with anti-CD3/anti-CD28-coated beads cultured with or without pulverized ECM; (**C**) Representative plots and (**D**) quantitation of Annexin-V and 7-AAD expression as a measure of apoptosis/necrosis in human naïve CD4^+^ T cells stimulated with anti-CD3/anti-CD28-coated beads cultured with or without ECM; (**E**) Representative plots and (**F**) quantitation of FoxP3 expression in CD4^+^CD25^+^ cells converted from human naïve CD4^+^ T cells stimulated with anti-CD3/anti-CD28-coated beads and IL2 cultured with or without ECM (with permission from Peloso *et al.* *Ann Surg* 2015, ahead of print) [145]. * denotes *p* < 0.05.

Currently, protocols for generation of the ECM scaffolds from transplantable organs are well established in rat, mouse, porcine and human models. Scaffolds that preserve the architecture and function of the organ can be successfully produced and are being used as a template for organ bioengineering and regeneration. Interestingly enough, ECM scaffolds show remarkable immunomodulatory properties, as recently demonstrated by Orlando *et al.* in the human pancreatic ECM model [145]. Following promising results with the porcine model, the decellularization of the human pancreas allowed production of ECM scaffolds that, when pulverized, are able not only to mitigate lymphocyte proliferation, as previously shown, but also to induce a T-Reg phenotype in T cells (Figure A2) [146,147,148]. To achieve further advancements in this area of tissue regeneration, the studies reviewed highlight the need for optimizing the recellularization process, and differentiation and expansion of the necessary cells types from clinically feasible sources.

## 6. Summary

Regenerative medicine is a growing field that offers novel solutions to wound repair, tissue regeneration, and organ transplantation. Tissue engineering (TE) is a process by which cellular growth on ECM scaffolds repairs, replaces, and regenerates organs or tissues with impaired function. The biomaterials used in TE enable the success of these processes, but also present challenges to graft viability.

Natural polymer scaffolds are advantageous in terms of biomimeticity and structural complexity. These naturally occurring materials, however, lack controlled degradation and have the potential to be immunogenic. Numerous factors of synthetic materials including functionality, mechanical strength, and degradation can be controlled by manipulating chemical and physical properties during manufacturing. In general, both natural and synthetic polymer-based scaffolds are more efficacious when conjugated or copolymerized with another material. Resultant composite scaffolds consisting of two or more polymers convey properties of each material present, which allows for compensation of disadvantages of coexisting polymers. Copolymerization also allows for modulation of properties via polymer ratio control.

Decellularized organs are a potential solution to mitigating the waste of donated organs deemed unsuitable for transplantation. Decellularization has shown some success in several *in vitro* studies of lungs, kidneys, and livers. Survival of grafts originating from decellularized scaffolds *in vivo* has been problematic. Although highly biomimetic, decellularized scaffolds are currently not as viable as natural and synthetic polymer scaffolds because of rejection after transplantation.

Constructing a viable graft depends, in part, on choice of scaffold material. Biomaterials comprise a vast group of synthetic and naturally occurring substances that can be shaped into structures of immense complexity that convey specific function. Variation of chemical and physical properties ensures biomaterials are more or less suitable to serve as scaffolds for TE of specific organs or tissues. Therefore, there is as of yet no particular universally optimal biomaterial.
